# A Measurement Model of Mutual Influence for Information Dissemination

**DOI:** 10.3390/e22070725

**Published:** 2020-06-30

**Authors:** Liang Zhang, Yong Quan, Bin Zhou, Yan Jia, Liqun Gao

**Affiliations:** College of Computer, National University of Defense Technology, Changsha 410073, China; gfkdzliang@gmail.com (L.Z.); qy8801@nudt.edu.cn (Y.Q.); gaolinudt@gmail.com (L.G.)

**Keywords:** mutual influence, Survival Analysis, information dissemination, propagation probability, TMIVM (Topic Mutual Influence Vector Model)

## Abstract

The recent development of the mobile Internet and the rise of social media have significantly enriched the way people access information. Accurate modeling of the probability of information propagation between users is essential for studying information dissemination issues in social networks. As the dissemination of information is inseparable from the interactions between users, the probability of propagation can be characterized by such interactions. In general, there are differences in the dissemination modes of information that carry different topics in a real social network. Using these factors, we propose a method (TMIVM) to measure the mutual influence between users at the topic level. The method associates two vectorization parameters for each user—an influence vector and a susceptibility vector—where the dimensions of the vector represent different topic categories. The magnitude of the mutual influence between users on different topics can be obtained by the product of the corresponding elements of the vectors. Specifically, in this article, we fit a social network historical information cascade data through Survival Analysis to learn the parameters of the influence and susceptibility vectors. The experimental results on a synthetic data set and a real Microblog data set show that this method better measures the propagation probability and information cascade predictions compared to other methods.

## 1. Introduction

Efficiently acquiring and disseminating valuable information, thereby, revealing the mechanisms and rules of information dissemination in social networks, has become the research focus of scholars. At present, the existing achievements mainly involve the fields of influence maximization [1,2,3], forwarding behavior prediction [4,5], social recommendations [6,7], viral marketing [8,9], and so on. Modeling and measuring the probability of information dissemination among the users of social networks provides an essential basis for such research. A relatively simple method is to set the propagation probability for social relations in the study of an information cascade, and use various propagation models to simulate the process of information dissemination [10]. This type of method has apparent limitations: Predicting the propagation path of information is based on a pre-set propagation probability, which can easily cause large deviations. The information dissemination effect is different for different users, so it is difficult for researchers to set a reasonable value for each propagation probability in advance. Another more effective method is to construct a probability generation model for information dissemination based on historical data. The basis for the probability generation model to calculate the propagation probability is determined by the historical data of information cascades. If there is no interaction between users in the previous data, then the model will overfit when it performs its parameter inference. That is, the propagation probability between the users will be 0, which means that no information can be transmitted between these two users in the future. This is obviously inconsistent with an actual situation. In the real world, users will have different preferences for different topics. Furthermore, the information dissemination patterns of different topics in social networks are also different, such that the mutual influence between users will be different for different topics [11]. Based on the above factors, this paper proposes the TMIVM (Topic Mutual Influence Vector Model), which calculates the mutual influence between social network users in the topic dimension. Specifically, the TMIVM associates two vectors for each user namely, an Influence vector and a Susceptibility vector. The influence vector indicates the degree to which the user can influence others, while the susceptibility vector indicates the degree to which the user is influenced by others. The dimension of the vector represents the different topic categories. In this way, under different topics, the mutual influence between users can be calculated by the product of the corresponding elements of the influence vector and the susceptibility vector. The main contributions of this article are as follows:

1. A new topic-related mutual influence calculation model, TMIVM, is proposed. The characteristics of this model use the vectorization method to express a user’s influence and susceptibility. Historical information cascade data are used for parameter training without calculating too many variables, significantly improving calculation efficiency.
2. An information propagation probability calculation method, based on Survival Analysis and TMIVM, is designed. This method fully considers the textual content of information and the time-series model of information dissemination. Survival Analysis is used to construct the likelihood function of cascade propagation, and the parameters are inferred by gradient descent.
3. Many experiments are carried out on synthetic and real data sets. The results demonstrate the feasibility of the information propagation probability measurement method based on the TMIVM. Compared to other comparison methods, the performance of our method is better in terms of its information cascade prediction, among other aspects.

## 2. Related Work

Existing research has focused on establishing an information propagation probability variable for the social relationships between users, followed by estimating that variable using the information of the underlying structure of the social network, information cascade records, and user attributes. For example, Kempe et al. used network topology (degree and centrality) to measure the probability of information transmission among users in an independent cascade propagation model when studying the problem of influence maximization [2]. Java et al. evaluated the efficiency of the influence model in a blog network and proposed a method to calculate the propagation probability by blog forwarding frequency, concluding that selecting high-impact user groups can maximize the information dissemination effect [10]. Yang et al. constructed a linear influence model in the process of studying the predictions of the popularity of twitter blog information and designed a time-dependent user influence function, based on the assumption that information dissemination is dominated by user influence [12]. To solve the problem of predicting the propagation probability in complex networks, Saito et al. used the node infection probability model to fit the information cascade data in an independent cascade model, and calculated the posterior propagation probability value via the EM (Expected Maximum) method [13]. Artzi et al. studied the forwarding behavior of users, calculating the probability of users affected according to the content of blog posts and user attributes [14]. Xiong et al. put forward a SCIR model of information dissemination based on an infectious disease model (SIR). The authors considered that a user’s browsing information without forwarding is the R state, and the probability of infection between users is related to the infected users with the same degree density [15]. These methods aimed to study issues related to the dissemination of information in social networks and did not focus on the role of user interactions in disseminating information. The present article considers the user’s point of view, combined with user interaction behavior and information cascade data, to construct a propagation probability calculation method based on mutual influence.

To accurately measure the propagation probability of information or mutual influence, relevant scholars have conducted a great deal of empirical research and model verification. Some have proposed various methods that are similar to the work in this article. Myers explored the potential information dissemination network structure of social networks and proposed a CONNIE model that uses convex programming and heuristic methods to infer the prior probability for each infection edge [16]. Gomez-Rodriguez et al. proposed the NETINF model, which uses sub-module optimization features to obtain an optimal network connectivity structure [17]. Both of these methods are probabilistic generative models, and both assume that the propagation probabilities in social networks are predefined constants, which affects the efficiency of the model. Based on this, Gomez-Rodriguez et al. improved their model and proposed a continuous-time model, NETRATE, based on the survival model. By maximizing the likelihood function of the observed data, the propagation probability of the information among users was inferred [18]. Later, to improve the accuracy of information propagation predictions, Gomez-Rodriguez et al. constructed additive and multiplicative risk functions in the survival model and experimentally confirmed that the new method is more effective [19]. Du et al. suggested that the dissemination patterns of different types of topic information are different; combined with the topic attributes of that information, the authors proposed the TOPICCASCADE model to calculate topic-related propagation probability [11]. Wang et al. constructed the influence and susceptibility vectors with latent variables for users and proposed a LIS model, based on user behavior time-series information in cascade data. Their experimental results on real data sets proved that the model has good performance in its propagation predictions and other aspects [20]. Most of these methods are based on edge modeling in a communication network, while the LIS model is based on the influence and susceptibility of users. The latter requires only a few parameters, and its calculation efficiency is high, thus providing a reference for the construction of the model in this paper.

In the modeling process, we use Survival Analysis to build a cascade likelihood function. Before that, let us briefly introduce Survival Analysis.

***Survival Analysis.*** Survival Analysis is the theoretical basis of this paper’s method and is a subject that studies the statistical phenomena of survival phenomena and response time data [21]. It is mainly used for statistical analysis of the expected duration of one or more events, such as the death of biological organisms or malfunctions in machine systems. In [18], the idea of Survival Analysis was first introduced in the context of studying information dissemination in social networks and achieved good experimental results. First, define a non-negative continuous random variable T, which represents the time of an event. Then, if f(t) represents the probability density function of the random variable T and F(t) is the cumulative distribution function representing the random variable T, it follows that $F\left(t\right)=P\left(T\leq t\right)=\int_{0}^{\infty }f\left(u\right)\mathrm{du}$. The survival function S(t) represents the probability that the event has not occurred until time t, as defined below,

(1) $$S\left(t\right)=P\left(T\geq t\right)=1-F\left(t\right)=\int_{t}^{\infty }f\left(u\right)\mathrm{du},$$

where S(t) is a monotonically decreasing continuous function, so S(0)=1 and $S\left(\infty \right)=\lim_{t\to \infty }S\left(t\right)=0$, which are the boundary conditions.

The hazard function, h(t), is the basic function in Survival Analysis, which reflects the risk of death of the research object at a certain moment; that is, the event does not occur until time t but occurs in the time interval [t, t +Δt). The probability, Δt, is an infinitesimal amount of time t. Given S(t) and F(t), h(t) can be calculated by

(2) $$h\left(t\right)=\underset{\Delta t\to 0}{\lim}\frac{P(t\leq T\leq t+\Delta t|T\geq t)}{\Delta t}=\frac{P\left(t\leq T\leq t+\Delta t\right)}{\Delta t}\cdot \frac{1}{P\left(T\geq t\right)}=\frac{f\left(t\right)}{S\left(t\right)}.$$

The above equation uses the Bayesian principle P(A∩B)=P(A)·P(B|A). To find the difference between the two sides of Formula (1), we can take f(t)=−S′ (t) and include Formula (2) to obtain the first-order linear ordinary differential equation, −S′(t)+h(t)·S(t)=0, with respect to the survival function S(t). Combined with the boundary condition, S(0)=1, both S(t) and f(t) can then be expressed as a function of h(t):

(3) $$\ln S\left(t\right)=-\int_{0}^{t}h\left(u\right)\mathrm{du},\;f\left(t\right)=h\left(t\right)S\left(t\right).$$

Therefore, when the expression of any one of the survival function S(t), the hazard function h(t), or the probability density function f(t) is known, the expressions of the other two functions can be derived. This provides a theoretical basis for the construction and simplification of the method presented below.

## 3. Proposed Model: TMIVM

We will first provide several related definitions, as shown in Table 1.

In general, a social network can be defined as a directed graph model G=(V, E), where V represents a set of users, and E represents a set of directed edges. For any pair of users u, v∈V, if there is a directed edge, then (u, v)∈E; if not, then (u, v)∉E. The edges in the social network are formed by the social relationships between users, which may be relationships defined by following, forwarding, or friends. In this context, E refers to a collection of following relationships. Due to the characteristics of the social platform system in actual use, information will be spontaneously pushed to a user’s followers, causing the information to spread along the underlying network.

**Figure 1:**
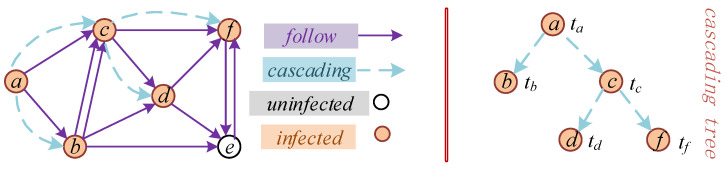
Schematic diagram of the information cascade propagation.

We use the symbol $C^{m}$ to represent the cascade records caused by information m among users. According to the previous analysis, these records mainly include three aspects: user, infection time, and cascading tree structure. Clearly, a piece of information produces a cascading record. Formally, the mathematical method represents the cascade records as a set of triples: $C^{m}=\left\{\left(u_{1}^{m},{\;t}_{1}^{m},{\;P}_{≼u_{1}}^{m}\right),\;\left(u_{2}^{m},{\;t}_{2}^{m},{\;P}_{≼u_{2}}^{m}\right),\ldots ,\;\left(u_{N}^{m},{\;t}_{N}^{m},{\;P}_{≼u_{N}}^{m}\right)\right\},\;1\leq i\leq N$, where $t_{i}^{m}$ represents the time at which user $u_{i}^{m}$ forwards the information, and $P_{≼u_{i}}^{m}$ represents the parent user of user $u_{i}^{m}$ in the cascading tree; that is, the user who affects their forwarding. The scale of the cascade record $C^{m}$ refers to the total number of users who forward this information in information dissemination, represented by $\left|C^{m}\right|$. Therefore, in a cascade record, the set $\left\{P_{≼u_{1}}^{m},{\;P}_{≼u_{2}}^{m},\ldots ,{\;P}_{≼u_{N}}^{m}\right\}$ represents a tree structure built by forwarding the relationships between users. As there is only one publisher of the original information, if $u_{j}^{m}$ is the root node in the cascade tree, then $t_{j}^{m}=\min\left\{t_{1}^{m},\ldots ,t_{N}^{m}\right\}$ is satisfied, indicating the release of the original information m. The time is $m_{t}=t_{j}^{m}$, the publisher is $m_{u}=u_{j}^{m},$ and $P_{≼u_{N}}^{m}=\emptyset$, 1≤j≤N. In addition, if node $u_{j}^{m}$ is the cascade parent node of node $u_{i}^{m}$, then $P_{≼u_{i}}^{m}=u_{j}^{m}$ and $t_{j}<t_{i}$ are established, indicating that the time of forwarding the information must be later than the time at which the information is released. Taking Figure 1 as an example, the specific type of information is not considered at present, and the cascade records are $C=\left\{\left(a,{\;t}_{a},\;\emptyset \right),\;\left(b,{\;t}_{b},\;a\right),\;\left(c,{\;t}_{c},\;a\right),\;\left(d,{\;t}_{d},\;c\right),\;\left(f,{\;t}_{f},\;c\right)\right\}$; a is the root node, $t_{a}=\min\left\{t_{a},t_{b},t_{c},t_{d},t_{f}\right\}$. As $P_{≼f}=c,{\;t}_{c}<t_{f}$ holds.

**Mutual influence model.** Establishing a mutual influence model for social network users is the focus of this work. We model two K-dimensional non-negative vectors for each user: an influence vector $I_{u}=\left(I_{u}^{1},I_{u}^{2},\ldots ,I_{u}^{K}\right)$ and a susceptibility vector $S_{u}=\left(S_{u}^{1},S_{u}^{2},\ldots ,S_{u}^{K}\right)$, where $I_{u}^{k}\geq 0$, $S_{u}^{k}\geq 0$, ∀1≤k≤K. The larger the value of an element in $I_{u}$ or $S_{u}$ is, the higher the degree to which the user influences or is influenced by others, respectively. *K* represents the number of topics, indicating that the user’s influence and susceptibility are different in different topics. In this way, the mutual influence ${\mathrm{MI}}_{u\to v}$ between user u and user v can be expressed as,

(4) $${\mathrm{MI}}_{u\to v}=I_{u}\circ S_{v}=\left(I_{u}^{1}\cdot S_{v}^{1},I_{u}^{2}\cdot S_{v}^{2},\ldots ,I_{u}^{K}\cdot S_{v}^{K}\right),$$

where ${\mathrm{MI}}_{u\to v}\geq 0$**,** and ∘ is the Hadamard product of the vector, which represents the product of the corresponding elements of the vector [22]. By definition, the mutual influence is asymmetric, that is, ${\mathrm{MI}}_{u\to v}\neq {\mathrm{MI}}_{v\to u}$. Unlike existing models, this model characterizes the mutual influence from the user’s point of view, instead of simply setting a weight coefficient for the relationship edge of the underlying network structure. The approach in the literature suggests that the influence of different user pairs is independent. However, this assumption is not scientific enough because the interactions associated with the same user are clearly related to the degree of influence or susceptibility of the user. In consideration of this, when using Formula (4) to calculate the influence on other users, the influence vector $I_{u}$ is used, and the susceptibility vector $S_{u}$ is likewise applied to calculate the degree of influence by other users. This user-centered calculation can effectively associate the influence between different user pairs, which is consistent with the modeling motivation in the literature [23]. The mutual influence ${\mathrm{MI}}_{u\to v}$ between users is also a k-dimensional non-negative vector. The element $I_{u}^{k}\cdot S_{v}^{k}$ represents the influence of user u on user v with respect to topic K.

**Calculation of propagation probability.** In the process of modeling information propagation in social networks based on Survival Analysis, the non-negative continuous random variable t is instantiated by combining the information propagation process. In social networks, information spreads like a virus. Users who publish original information are like infection sources. Users who forward information are equivalent to the infected. Users who have not forwarded the information are immune. Therefore, in this paper, we regard the time interval for users to forward information as a random event of the variable T, T∈[0,+∞). Thus, the survival function S(t) can be interpreted as the probability that a user has not forwarded the information until time t, and the hazard function h(t) can be interpreted as the risk coefficient of a user who has not yet forwarded the information at time t. Therefore, the cumulative distribution function F(t) can be interpreted as the probability that a user will forward information within the interval spanning from 0 to t. Here, we first need to define the probability of information propagation between users, which is related to the topic of the information and the interaction between users, as follows,

(5) $$D_{u\to v}^{m}={\mathrm{MI}}_{u\to v}^{\prime }\cdot m_{o}=\sum_{k}I_{u}^{k}\cdot S_{v}^{k}\cdot m_{o}^{k},$$

where $D_{u\to v}^{m}\geq 0$, ${\mathrm{MI}}_{u\to v}^{\prime }$ represents the transposition of the vector, $m_{o}$ is the topic distribution vector of information m, and the different elements of the vector represent the information’s propensity under different topics. The definition of Formula (5) shows that the topic content of information has an influence on the dissemination of that information among users and that popular information is more likely to induce social network users to spread and discuss it. In addition, the formula also implies an implicit condition $D_{u\to v}^{m}\neq 0$ that holds if and only if the information that user u has forwarded is visible to user v.

**Figure 2:**
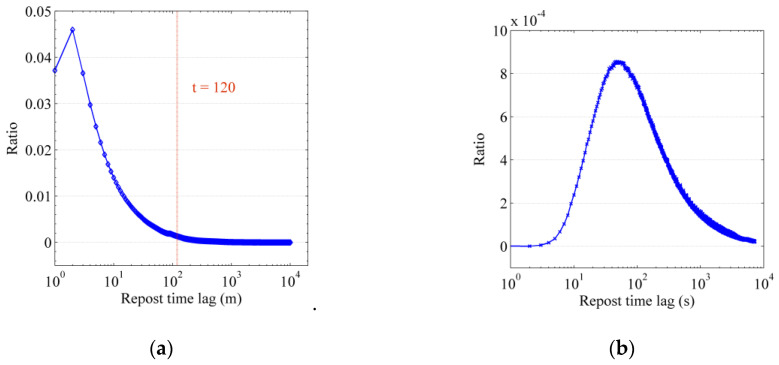
Distribution statistics of the time interval between the original blog posts being forwarded. (**a**) Distribution of forwarding time intervals within one week; (**b**) Distribution of forwarding time intervals within 2 h.

***The likelihood function of cascade records.*** In the Sina Weibo data collected in this paper, there are 2,749,631 cascade records triggered by original blog posts, where the distribution of the forwarding interval is shown in Figure 2. Figure 2a shows the probability distribution of the forwarding time within one week. It can be seen that the forwarding probability increases first and then decreases. Figure 2b shows the forwarding probability distribution of the time interval within 120 min in Figure 2a, which more clearly illustrates the effect of the forwarding probability increasing first and then decreasing. According to the results of the data analysis, the Rayleigh model can better capture the above distribution rule of the forwarding time interval. In this paper, this model is used to instantiate the Survival Analysis. Then, the conditional probability density function of an uninfected user v affected by an infected user u at time $t^{m}$ who forwards the information m is as follows,

(6) $$f\left(t^{m}|t_{u}^{m},D_{u\to v}^{m}\right)=D_{u\to v}^{m}\cdot \left(t^{m}-t_{u}^{m}\right)\cdot \exp\left(-\frac{1}{2}\cdot D_{u\to v}^{m}\cdot {\left(t^{m}-t_{u}^{m}\right)}^{2}\right),$$

where exp(⋅) is the exponential function with the natural constant *e* as the base. Formula (6) reflects the distribution law of the time interval of a user forwarding information, as shown in Figure 2. Then, at time $t^{m}$, the conditional hazard function of an infected user u for an uninfected user v related to information *m* is as follows:

(7) $$h\left(t^{m}|t_{u}^{m},D_{u\to v}^{m}\right)=D_{u\to v}^{m}\cdot \left(t^{m}-t_{u}^{m}\right).$$

Formula (7) shows that the risk of infected users to uninfected users increases with time. When the user cannot browse to information m through u (meaning that the danger does not exist), then $h\left(t^{m}|t_{u}^{m},D_{u\to \cdot }^{m}\right)=0$. Similarly, under the influence of an infected user *u*, the probability that user v will not forward the information *m* in time $t^{m}$ is:

(8) $$S\left(t^{m}|t_{u}^{m},D_{u\to v}^{m}\right)=\exp\left(-\frac{1}{2}\cdot D_{u\to v}^{m}\cdot {\left(t^{m}-t_{u}^{m}\right)}^{2}\right).$$

In summary, Formulas (6)–(8) are the probability density function, hazard function, and survival function in Survival Analysis, respectively. In this paper, the time interval variable *t* of a user forwarding information is expressed concretely. The definitions of these formulas are all related to the specific information, *m*, and the propagation probability, which indicates that the method in this paper focuses on the information content attributes in the propagation process. It is worth noting that the calculation of these formulas is not related to the specific time of information publishing or forwarding but only to the time difference between user behaviors.

In this paper, the recorded data on a particular information cascade in a social network in the experiment are limited to a specific period $\left[0,{\;T}_{w}^{m}\right]$, which is called the observation period of information m. During the observation period, the cascade record contains two types of users: infected and uninfected. The uninfected users are not the same as those who never forward a message but are simply recorded as not forwarding a message during the observation period. To simplify the computational complexity (∀m, making $T_{w}^{m}=T_{w})$, all information cascades adopt the same observation period.

For the uninfected users, $u_{i}^{m}\in {\mathrm{NU}}^{m}$, it is necessary to calculate the probability that the users will not forward the information until the end of the observation time. ${\mathrm{NU}}^{m}$ refers to the set of users who do not forward information in the cascade of m, also known as negative examples. As each infected user can independently affect other uninfected users, the survival probability of uninfected users in the cascade record of *m* is determined by the product of the relevant survival function of each infected user, as follows,

(9) $$E_{u_{i}^{m}}^{-}\left(C^{m}\right)=\prod_{u_{j}^{m}\in \Omega_{i}^{m}}S\left(T_{w}|t_{j}^{m},D_{j\to i}^{m}\right),$$

where $\Omega_{i}^{m}$ represents the set of users whose forwarding information is visible to $u_{i}$ during the propagation of information m. For example, $\Omega_{e}^{m}=\left\{b,\;d,\;f\right\}$ of user e is not infected in Figure 1. $\Omega_{i}^{m}$ can also be ∅, which means that the user $u_{i}^{m}$ has neither forwarded the information nor browsed m through other users.

For the infected users, $u_{i}^{m}\in {\mathrm{PU}}^{m}$, it is necessary to calculate the probability that the users will be influenced by other users during the observation time and forward the information. ${\mathrm{PU}}^{m}$ refers to the set of users who forward information m in the cascade, also known as positive examples. In the cascade record, every infected user has a clear cascade path, such that the probability of forwarding information is determined by the parent node in the cascade tree. However, the previous assumption is that users can only be infected once, and the probability of surviving without being infected when seeing other users forwarding information should also be calculated,

(10) $$E_{u_{i}^{m}}^{+}\left(C^{m}\right)=f\left(t_{i}^{m}|t_{≼i}^{m},D_{≼i\to i}^{m}\right)\cdot \prod_{q\neq ≼i,{\;u}_{q}^{m}\in \Omega_{i}^{m}\cap t_{q}^{m}<t_{i}^{m}}S\left(t_{i}^{m}|t_{q}^{m},D_{q\to i}^{m}\right),$$

where ≼i is a simplification of $P_{≼u_{i}}^{m}$, representing the parent node of user $u_{i}$ in the cascade tree constructed by the propagation record of m. The first item of Formula (10) shows that infecting the parent node ≼i has a direct impact on $u_{i}^{m}$ forwarding information at time $t_{i}^{m}$, while the second item shows that, in addition to node ≼i, the user is also affected by other users but will not be affected by other users when facing the same information after infection. Using the transformation relation f(t)=h(t)S(t), Formula (10) can be written as follows:

(11) $$E_{u_{i}^{m}}^{+}\left(C^{m}\right)=h\left(t_{i}^{m}|t_{≼i}^{m},D_{≼i\to i}^{m}\right)\cdot \prod_{{\;u}_{q}^{m}\in \Omega_{i}^{m}\cap t_{q}^{m}<t_{i}^{m}}S\left(t_{i}^{m}|t_{q}^{m},D_{q\to i}^{m}\right).$$

In any cascade record, except for the root node user who publishes the original information, all other users are either infected or uninfected; that is, ${\mathrm{NU}}^{m}\cup {\mathrm{PU}}^{m}=V$, ${\mathrm{NU}}^{m}\cap {\mathrm{PU}}^{m}=\emptyset$. For an information cascade record, $C^{m}$, the likelihood function can be expressed as the product of the probability of all users who are infected or uninfected:

(12) $$E\left(C^{m}\right)=\prod_{u_{i}^{m}\in {\mathrm{PU}}^{m}}E_{u_{i}^{m}}^{+}\left(C^{m}\right)\times \prod_{u_{i}^{m}\in {\mathrm{NU}}^{m}}E_{u_{i}^{m}}^{-}\left(C^{m}\right).$$

Introducing Formulas (9) and (11), we obtain:

(13) $$E\left(C^{m}\right)=\prod_{u_{i}^{m}\in {\mathrm{PU}}^{m}}h\left(t_{i}^{m}|t_{≼i}^{m},D_{≼i\to i}^{m}\right)\cdot \prod_{u_{q}^{m}\in \Omega_{i}^{m}\cap t_{q}^{m}<t_{i}^{m}}S\left(t_{i}^{m}|t_{q}^{m},D_{q\to i}^{m}\right)\cdot \prod_{u_{i}^{m}\in {\mathrm{NU}}^{m}}\prod_{u_{j}^{m}\in \Omega_{i}^{m}}S\left(T_{w}|t_{j}^{m},D_{j\to i}^{m}\right).$$

Generally, information cascade data C contain multiple cascade records $\left\{C^{1},C^{2},\ldots ,{\;C}^{M}\right\}$, where M represents the total amount of information. Different information causes different cascade records, and these cascade records are independent of each other. Therefore, the likelihood function of the cascade data is the product of the likelihood functions of all individual cascade records:

(14) $$E\left(C^{m}\right)=\prod_{u_{i}^{m}\in {\mathrm{PU}}^{m}}h\left(t_{i}^{m}|t_{≼i}^{m},D_{≼i\to i}^{m}\right)\cdot \prod_{u_{q}^{m}\in \Omega_{i}^{m}\cap t_{q}^{m}<t_{i}^{m}}S\left(t_{i}^{m}|t_{q}^{m},D_{q\to i}^{m}\right)\cdot \prod_{u_{i}^{m}\in {\mathrm{NU}}^{m}}\prod_{u_{j}^{m}\in \Omega_{i}^{m}}S\left(T_{w}|t_{j}^{m},D_{j\to i}^{m}\right).$$

Based on Formula (14), the research problems in this paper can be described as follows:

***Problem definition:*** Given social network cascade data G=(G, C), where G=(V, E) represents the social network structure and users, and $C=\left\{C^{1},C^{2},\ldots ,{\;C}^{M}\right\}$ is an information cascade record, our goal is to train a set of values of user influence vectors I and susceptibility vectors S to optimize the likelihood function value of C; that is, to solve the following optimization problems,

(15) $$\underset{I,S}{\mathrm{minimize}}-\ln E\left(C\right)+\lambda_{I}{\left\|I\right\|}_{F}^{2}+\lambda_{S}{\left\|S\right\|}_{F}^{2}\;{\mathrm{subject}\;\mathrm{to}\;I}_{u_{i}}^{k}\geq 0,{\;S}_{u_{i}}^{k}\geq 0,\;\forall k,\;i,$$

where $I=\left\{I_{u_{i}}^{k}\right\}\;\mathrm{and}\;S=\left\{S_{u_{i}}^{k}\right\}$. To avoid overfitting problems in the optimization process, regularization terms are introduced into the objective function, where $\lambda_{I}$ and $\lambda_{S}$ are the regularization factors, and ${\left\|\cdot \right\|}_{F}$ is the Frobenius norm. In this paper, the negative log-likelihood function of probability production is constructed by the mutual influence model, following which the parameters of the user influence and susceptibility vectors are estimated based on an optimization problem called TMIVM (Topic Mutual Influence Vector Model). The objective function of the optimization problem can be obtained by introducing Formulas (7) and (8) into (15):

(16) $$\tilde{E}\left(C\right)=-\sum_{m}\sum_{u_{i}^{m}\in {\mathrm{PU}}^{m}}(\ln\sum_{k}I_{u_{≼i}}^{k}\cdot S_{u_{i}}^{k}\cdot m_{o}^{k}\cdot \Delta_{i,≼i}^{m})+\sum_{m}\sum_{u_{i}^{m}\in {\mathrm{PU}}^{m}}\sum_{u_{q}^{m}\in \Omega_{i}^{m}\cap t_{q}^{m}<t_{i}^{m}}\frac{1}{2}\cdot \sum_{k}I_{u_{q}}^{k}\cdot S_{u_{i}}^{k}\cdot m_{o}^{k}\cdot {\left(\Delta_{i,q}^{m}\right)}^{2}+\sum_{m}\sum_{u_{i}^{m}\in {\mathrm{NU}}^{m}}\sum_{u_{j}^{m}\in \Omega_{i}^{m}}\frac{1}{2}\cdot \sum_{k}I_{u_{j}}^{k}\cdot S_{u_{i}}^{k}\cdot m_{o}^{k}\cdot {\left(\Delta_{T^{w},j}^{m}\right)}^{2}+\lambda_{I}\sum_{u_{i}}\sum_{k}{\left(I_{u_{i}}^{k}\right)}^{2}+\lambda_{S}\sum_{u_{i}}\sum_{k}{\left(S_{u_{i}}^{k}\right)}^{2},$$

where $\Delta_{i,j}^{m}=t_{u_{i}}^{m}-t_{u_{j}}^{m}$. From the above analysis, we can see that this method only needs to solve $I_{u_{i}}^{k}$ and $S_{u_{i}}^{k}$. This user-centered influence vector model has a total parameter number of 2K|V|, while the modeling method with the user social side as the parameter needs to solve ${\left|V\right|}^{2}$ parameters. The scale of users in a social network is large (i.e., K≪|V|). Thus, solving fewer parameters can improve the effectiveness of this method.

## 4. Experiment and Result Analysis

We carried out many experiments on a synthetic data set and the real Sina Weibo data set to test and verify the performance of the method proposed in this paper compared to other similar methods. The experimental results demonstrate that, compared to other methods, the TMIVM-based method offers better performance in its information forwarding predictions, cascade scale predictions, user topic level interaction measurements, etc.

To accurately measure the propagation probability of information among users, some calculation methods based on mutual influence have been proposed by relevant scholars (see the discussion of relevant research in Section 2). In this paper, the following three representative methods were selected and compared to the proposed method in the experimental process to show its effectiveness. These three methods are as follows:

**ICEM method:** This method was proposed by Saito et al. and predicts the probability of information transmission in the independent cascade model [13]. This method considers that information in social networks is spread based on the underlying concern’s network structure, such that each propagation edge corresponds to a propagation probability $\kappa_{v,\;w}$, also known as the interaction between users. Based on this, combined with the independent cascade model of information dissemination, a probabilistic production model of cascade records is constructed. Finally, the parameters are learned by the expected maximum (EM) method,

$$\kappa_{v,w}=\frac{1}{\left|S_{v,w}^{+}\right|+\left|S_{v,w}^{-}\right|}\sum_{s\in S_{v,w}^{+}}\frac{{\hat{\kappa }}_{v,w}}{{\hat{P}}_{w}^{s}},$$

where $S_{v,w}^{+}$ indicates that the information is diffused from user v to the cascade record set of w, while $S_{v,w}^{-}$ indicates that user v has forwarded the information but w has not.

**NETRATE method:** This method infers the information propagation network and propagation probability through Survival Analysis [18] by assuming that the mutual influence between any two users can be represented by a scalar parameter $\alpha_{i,j}$. The hazard function is constructed based on the exponential, power-law, and Rayleigh distributions, respectively, to model the likelihood function for the time-series cascade data of users forwarding information, and the likelihood function is maximized to solve for $\alpha_{i,j}$ by using the convexity of the function. The experimental results in [18] show that the network structure inferred by the Rayleigh distribution and the interactions between users provides higher accuracy and better performance. In this paper, we used the NETRATE method based on Rayleigh distribution to carry out the comparative experiments.

**LIS method:** The main idea of this method is similar to that in this paper: modeling an influence vector and a susceptibility vector for each user [20]. However, this method does not consider the influence of information content on information dissemination. The influence vector and susceptibility vector are implicit variables, which do not show the weights of influence or susceptibility in different topics, instead associating a state variable with each user. In the process of information dissemination, the state variable of the user will change. By constructing the joint probability distribution of the state variable of the user, the influence vector and the susceptibility vector of the user can be solved.

The core idea of the TMIVM is to associate the influence vectors and susceptibility vectors of different topics for each user in a social network, while the ICEM and NETRATE methods build scalar parameters of mutual influence based on the relationships between users. To increase the comparability of the methods and prevent the overfitting problem in the above methods, we decomposed the scalar data into non-negative matrices according to the methods in [24]. For example, a certain method found the mutual influence $\alpha_{i,j}$ between $u_{i}$ and $u_{j}$. Through the matrix factorization technique $\alpha_{i,j}=\sum_{k}a_{k}^{i}b_{k}^{j}=a^{i}b^{j}$, where $a^{i}$ and $b^{j}$ are the influence vectors of $u_{i}$ and the susceptibility vectors of $u_{j}$, respectively.

### 4.1. The Synthetic Data Set

The synthetic data set in this paper refers to a directed graph generated by structural modeling to simulate a real social network. Based on the underlying simulation structure, different types of topic information are propagated using a simulation with preset parameters. The performance of the proposed method and the comparison methods was evaluated according to the propagation cascade records of the information. To verify the effectiveness of the method on user impact, susceptibility measurement, and information dissemination, we chose the widely used Forest Fire Model and a Kronecker Graph as the experimental objects. Through the social network analysis toolkit (SNAP) of Stanford University, we simulated and generated two directed graphs for the simulation experiments; namely, the Forest Fire Model, with 1024 nodes and 2084 directed edges, and the Peripheral Kronecker Graph(core-periphery [0.9, 0.5;0.5, 0.3]), with 1024 nodes and 2655 directed edges. A directed edge in the sampled graph structure data was considered to be a user’s attention relationship in the microblogging system. For example, the directed edge $\left(u_{i},{\;u}_{j}\right)$ indicates that user $u_{j}$ follows user $u_{i}$ and that user $u_{j}$ is a fan of $u_{i}$, such the information forwarded by $u_{i}$ is visible to $u_{j}$, which determines the direction of information flow (to a certain extent).

The key to the implementation of this method is to use the communication content as the input parameter of the algorithm. The topic distribution of the information content can distinguish the mutual influence of users on different topics. We used the LDA model to construct the topic distribution of the information and assumed that the number of topic categories of information in the composite data was K=6. In general, a user’s interest and preferences remain unchanged over a period of time. Therefore, for each user, a multi-dimensional variable $\vartheta_{i}\sim U{\left(0,\;1\right)}^{6}$ was randomly sampled to obtain the topic distribution of information released by the user at different times, where U(0, 1) refers to the uniform distribution between 0 to 1. To simplify the calculation, we chose the symmetric Dirichlet distribution to sample the topic distribution of information; that is, all elements in $\vartheta_{i}$ were equal. Next, using $\vartheta_{i}$ as a super-parameter of the Dirichlet distribution, we sampled the topic distributions of different information for different users in Dir($\vartheta_{i}$). In this way, we ensured that the topic information published by the same user was related, and avoided the information published by different users being different in their topic distribution. The information topic distribution obtained by sampling the Dir($\vartheta_{i}$) function was more in line with the actual situation. As the topic carried by one piece of information is relatively clear, it was unlikely to have a tendency on all topics and, generally, the distribution was focused on a few topic categories. To simplify the calculation, the topic distribution $m_{o}$ did not change throughout the whole process of information propagation.

Given the network structure data of the above synthetic Forest Fire Model and Kronecker Graph Model $G^{*}$, we also needed to construct cascade record data at different scales. First, we sampled the influence vector $I_{i}$ and the susceptibility vector $S_{i}$ for each user $u_{i}$, where $I_{i}\sim U{\left[0,\;1\right]}^{6}$, $S_{i}\sim U{\left[0,\;1\right]}^{6}$, and the values of different dimensions of the vector represent the influence and susceptibility of users with respect to different topics. That is, considering the elements of the influence and susceptibility vectors, 0 indicates that a user has no influence or is unlikely to be influenced by others on a topic, while 1 means that a user has the most influence or is the most likely to be influenced by other users with respect to a certain topic. Then, a user $u_{i}$ was randomly selected as the information publisher, becoming the root node user in the cascade tree. The topic distribution $m_{o}$ of the published information m was determined by the Dir($\vartheta_{i}$) sampling function. As the calculation formulas in the algorithm and the comparison algorithm in this paper do not depend on the absolute time of the user publishing or forwarding information, the time of the root node user publishing information was set as "0", thereby constituting the first data item $\left(u_{i},\;0,\;\emptyset \right)$ in the cascade record $C^{m}$ triggered by m. In the cascade communication, root node users affect their fans, and the information transmission path is controlled by the sampled attention relationship, while the infected fans, in turn, affect their fans, and the final information continues to spread. Based on the known cascade information *m*, according to the edge structure data and the defined parameter values in $G^{*}$, the breadth-first search method can be used to simulate the dynamics of information diffusion. It can be seen, based on the hypothesis, that every new infected (forwarding information) user has only one chance to infect their fans. The time of user infection is determined by the sampling in Formula (6). If a user is affected by multiple users and forwards information, the synthetic data set only needs to record the first time that it is successfully affected. The end of the cascade propagation process occurs when no new infected node appears or there is no new node forwarding information within the specified observation time window $T^{w}$. To simplify the calculation, the topic distribution $m_{o}$ was not changed throughout the whole process of information propagation.

Using the Forest Fire Model and Kronecker Graph, according to the data sampling method described above, we simulated three sets of information cascade data, including 1000, 2000, and 5000 cascade records. Each data set was randomly divided into five equal parts. We used the five-fold cross-validation method to evaluate the algorithm performance; that is, four parts were used for the training model, and the remaining part was used for the testing model. When $T^{w}=10$, the four different measurement models of mutual influence obtained different experimental results. Next, we compared and analyzed these experimental results.

**Figure 3:**
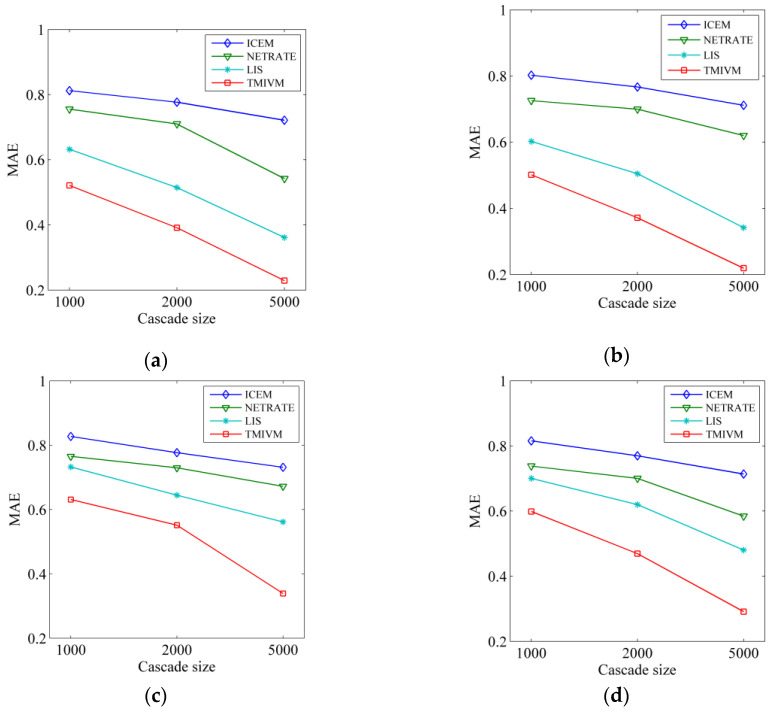
Evaluation of user influence and susceptibility measured in different data sets. (**a**) Estimation of influence in the Forest Fire Model; (**b**) Estimation of susceptibility in the Forest Fire Model; (**c**) Estimation of influence in the Kronecker Graph; (**d**) Estimation of susceptibility in the Kronecker Graph.

The user influence and susceptibility vector values were known in advance in the synthetic data set, and the four algorithms estimated these vector values based on the sampling data set. The accuracy of the algorithm was measured by the Mean Absolute Error (MAE). The MAE of the influence vector was calculated as follows:$\frac{1}{M}\sum_{u}\|I_{u}-I_{u}^{*}\|{}_{1}$, where M is the total number of users, $I_{u}$ is the influence vector value of sampling, $I_{u}^{*}$ is the influence vector value estimated by the algorithm, and ${\left\|\cdot \right\|}_{1}$ is the 1-norm of the vector. Similarly, the MAE index of the user susceptibility vector was obtained as shown in Figure 3. It can be seen in this figure that the performance trends of the four algorithms were similar in the different data sets. An increase of scale in the cascade data, resulted in a smaller MAE value, and the higher the accuracy of the algorithm. This is because large-scale cascade data improve the accuracy of algorithm parameter learning. For different data sets, the accuracy of the four algorithms in the Forest Fire model was significantly higher than that in the Kronecker graph, which may be due to the different structures of the underlying graph. No matter how the data were set, the MAE of the TMIVM algorithm proposed in this paper was the smallest, and its accuracy was higher for the inference of user influence and the susceptibility vector value, which demonstrates the effectiveness of this method in measuring user influence and susceptibility. At the same time, the measurement of user influence and susceptibility based on vector representations showed better performance, as the ICEM and NETRATE methods used edges for object modeling, as well as ultimately needing matrix decomposition technology to obtain the vector representations of user influence and susceptibility, thus reducing their accuracy. The results in Figure 3 also indirectly reflect the fact that it is difficult to quantitatively infer the influence and susceptibility of users. In this experiment, the MAE of the TMIVM algorithm, with the best performance, only reached about 0.3.

**Figure 4:**
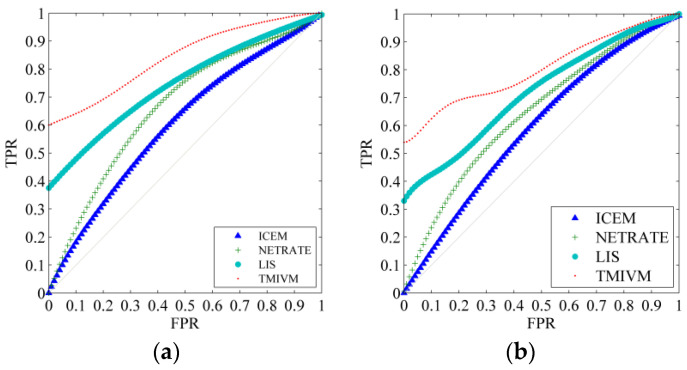
Algorithm prediction ROC curve of user forwarding information. (**a**) The ROC curve of the algorithm in Forest Fire Model; (**b**) The Roc curve of the algorithm in Kronecker graph.

The most direct application of this method is to predict the forwarding behavior of specific information to accurately measure the influence and susceptibility of users. The application scenario, in this paper, involves calculating the probability of whether a user will forward information according to Formula (11), given time *t* and the visible set of infected users Ω. We know the specific time when the user forwards the information; thus, the performance of the algorithm could be evaluated with this data as a reference. This is a typical classification problem. The probability of users forwarding information was successfully obtained, and the receiver operating characteristic curve (ROC) was used to measure the performance of the classifiers constructed by different algorithms, in order to indirectly explore the accuracy of user influence and susceptibility measurement. Figure 4 shows the ROC curves of the algorithms in the cascade record with a data scale of 5000. The abscissa is the false positive rate (FPR), that is, the proportion of users who are judged to forward information but actually have forward no information among all infected users. The ordinate is the true rate (TPR), which is the proportion of users who are judged to forward information who actually forward information among all infected users. The closer the ROC curve is to the upper left corner, the better the performance of the classifier. It can be seen that the TMIVM provided the best performance: When the FPR was 0, its TPR value could reach more than 54%. The performance of the LIS method was second: Its TPR value could exceed 33% when the FPR is equal to 0. ICEM and NETRATE were the worst, as they did not consider the topic of information and could not accurately predict the behavior of users forwarding information.

To explain the performance of user influence and susceptibility in forwarding prediction applications in detail, we calculated the AUC values and standard deviations of the four algorithms in different data sets, as shown in Table 2 and Table 3. The AUC is defined as the area under the ROC curve. The larger the value, the better the performance of the algorithm. In the 1000-scale data, the accuracy of the algorithm to predict user forwarding behavior was not high, and the standard deviation was also large because the amount of training data was small. Therefore, the accuracy of the model suffered. As the data size increased, the algorithm parameters were better calculated, the forwarding prediction became more accurate, and the standard deviation became smaller. As the LIS and TMIVM methods both consider user influence and susceptibility, the AUC values of these two methods were relatively high, and their accuracy was higher. Information content can directly affect a user’s forwarding behavior. Compared to the LIS method, using the topic hidden variable mechanism, the TMIVM method directly considers the information content in terms of user forwarding behavior predictions. Therefore, the AUC value in the forest fire model was increased by more than 10% in the proposed method. More importantly, the variance of the AUC value under the proposed method was the smallest in the different graph models and data sets at different scales, indicating that it was more stable.

In addition, we predicted the scale of the information cascade with different topic distributions. To simplify the experimental method, for each piece of information in the test data set, the original information publisher (root node user) was regarded as the initial infected user. The propagation probability of information among users was calculated by Formula (5); then, the propagation range of the information was simulated based on the independent cascade propagation model. Finally, the number of infected users was calculated. We analyzed the scale of communication according to topic categories, to reflect the impact of topic distribution on information dissemination in social networks. For each piece of information, the first three topic categories with the highest topic inclination were taken as the belonging topic categories in this experiment. The prediction results of the dissemination scale of this information were then considered as the experimental examples of these three belonging topic categories. This process not only enriched the number of experimental samples of the topic categories, but also conformed to the actual situation. That is, each piece of information could not cover all topic categories. By setting the first three topic categories, the test results of each piece of information were mapped to the three belonging topic categories.

**Figure 5:**
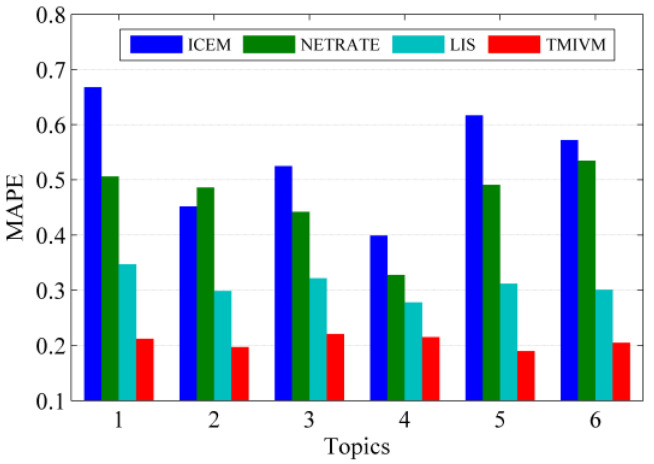
Cascading scale prediction of different types of topic information in the forest fire model.

The experimental results of the four algorithms in the forest fire model with a cascading scale of 5000 are shown in Figure 5. Here, in different topics, the prediction accuracy of LIS and TMIVM, based on vector representations, was higher and more stable. The MAPE value of the LIS method was less than the value of the six topics. The MAPE values were all around 0.2, indicating that the method of fusing topic distribution can predict the scale of information dissemination more effectively. The ICEM and NETRATE methods not only offered low prediction accuracy but also great performance differences under different topics. As these methods do not distinguish the topic attributes of information, their accuracy is not high when predicting the cascade size. In short, the mutual influence measurement model proposed in this paper can be effectively used to estimate the propagation probability of information in social networks. Further integration of the topic attributes of information could improve the accuracy of the model in cascading scale prediction.

**Figure 6:**
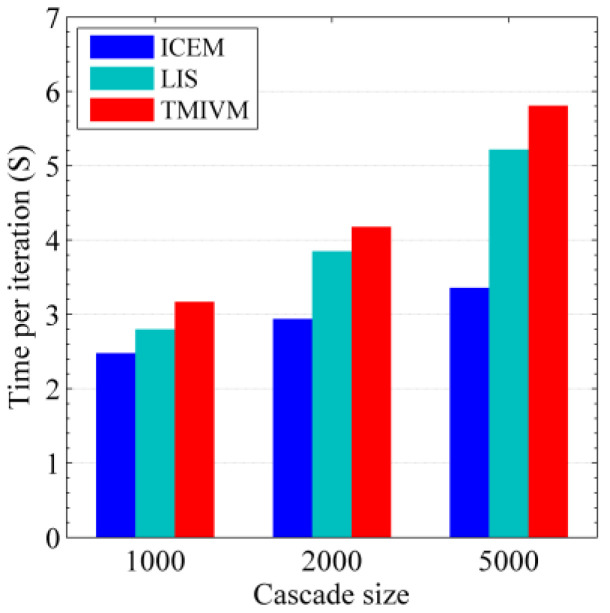
Operational efficiency of the algorithm in the Kronecker graph model.

Figure 6 shows the computational efficiency of the algorithm for solving user influence and susceptibility in the forest fire model. The NETRATE algorithm only needed to be run once for each node to obtain the influence value between all nodes, and the other three methods are iterative algorithms. Therefore, the former did not participate in the calculation efficiency comparison. This time is the average time required for the core iteration steps of the algorithm, excluding the time required for data preprocessing and the subsequent matrix decomposition. It can be seen that the iterative calculation efficiency of the ICEM method was more efficient. This is because there were only 2655 edges in the Kronecker graph model; therefore, the algorithm only needed to solve 2655 parameters, and each E-M estimation does not cost much time. The LIS and TMIVM methods needed to solve 2048 6-dimensional vector parameters, each iteration required solving the gradient function and step size update, which took more time. However, the results of the LIS and TMIVM methods were not subject to matrix factorization. In contrast, the TMIVM method was the least efficient, as it uses the Adagrad method to determine the learning rate; therefore, it took slightly more time than the LIS method.

### 4.2. Real Data Set

The real data set in this paper comes from Sina Weibo, the largest microblog service platform in China. The collection method mainly involved selecting 100 users as seed users to crawl microblog data according to their activity on the platform. Finally, we obtained a real data set of 174,206 users and 23,846,644 microblogs, including 9,234,475 original microblogs. Microblog data include the publishing time, user information, forwarding content, original blog information, etc.

Through heuristic data filtering methods, we obtained a relatively complete cascading data set on the real Weibo platform, involving a total of 10,794 users and 2,949,798 pieces of Weibo data, as shown in Table 4. Obviously, in the real data set, the number of users was much smaller than the number of relationships formed between users; on average, each original Weibo user is forwarded 50.4 times, and the largest cascade record triggered the forwarding behavior of 1158 users. During the experiment, the data in Table 4 were randomly divided into 10 parts, and the performance of all algorithms was tested using the 10-fold cross-validation method. During the experiment, the method proposed in this paper used the topic features of the Weibo questions. Therefore, the textual content of 57,347 original Weibo posts was calculated using the classic LDA model [25]. At this point, the topic category parameter was set to K = 20.

**Figure 7:**
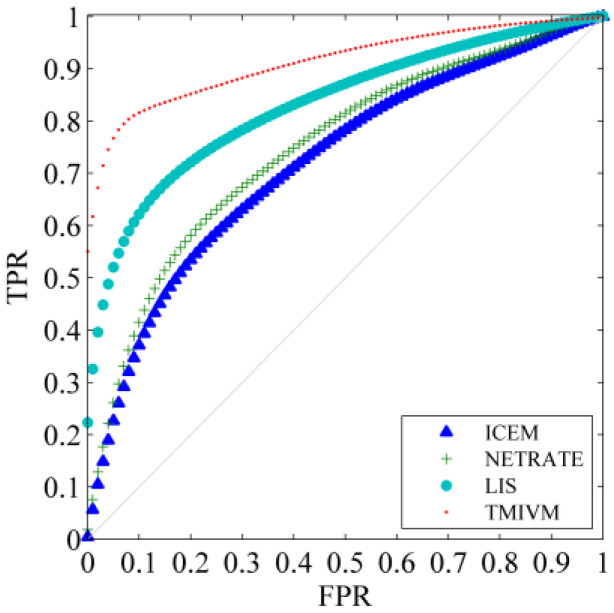
Information forwarding cascade prediction: ROC curve.

Compared to synthetic data, in real data sets, because the user’s influence and susceptibility cannot be determined in advance, we directly used the forwarding cascade prediction of information to test the performance of the algorithm; that is, the known social network G =(V, E), V =10,794, | E |=341,794, and the original Weibo *m* forwarding record before time *t*—the algorithm needed to speculate the probability of a user forwarding *m* at time *t*, where *t* is the time when the user actually forwarded the information observed in the real data set. Similarly, the ROC curve was used to compare the performance of different algorithms, as shown in Figure 7.

Compared to Figure 4, for the cascade prediction problem of Weibo, the results of the four algorithms in the Weibo data set were better than those in the synthetic data set. This is because the cascade propagation dynamics of information in Sina Weibo are more in line with the assumptions of various methods. For example, users will publish microblogs with consistent topic distributions according to their own interests. Users will only pay attention to other users who are interested, and the information propagation path will thus be more reasonable. For the TMIVM method, when the true rate reached 80%, its false-positive rate was only about 8%, indicating that the method has a strong ability to discriminate user forwarding behavior.

**Figure 8:**
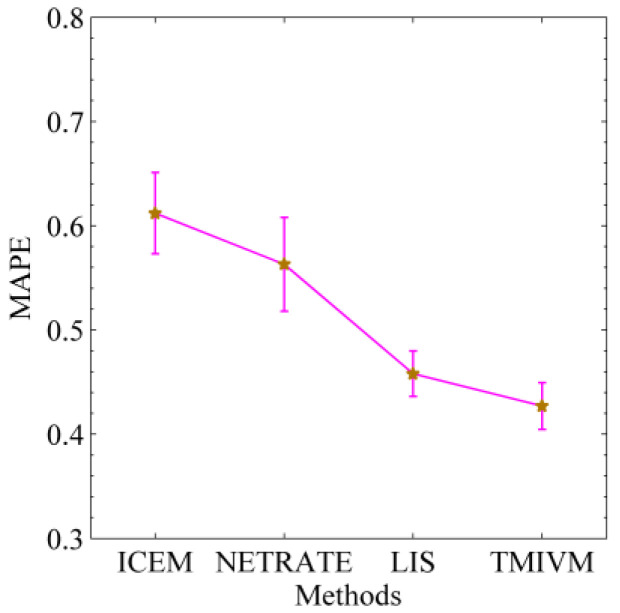
Prediction of an intermediate scale real data set.

Figure 8 shows the ability of the four algorithms to predict the cascade of information in the Weibo data set. The smaller the MAPE value is, the better the performance of the method in predicting the cascade size. It can be seen from the figure that the MAPE values of all algorithms were above 40%. Compared to the synthetic data set, the scale of the microblog cascade is uneven, which introduces challenges for accurate predictions. The relationship of concern between users is not static and may change at any time, which makes it difficult to capture the underlying structure of information dissemination and can easily affect the simulation of information dissemination. In general, the LIS and TMIVM methods, based on user influence and susceptibility, were superior to the ICEM and NETRATE methods in modeling the probability of edge propagation between users. In addition, the latter two methods were also poorly documented, as they over-fit the cascading data set, resulting in the inaccurate calculation of propagation probabilities between users. This reflects the rationality of using mutual influence to calculate the propagation probability of information between users.

**Figure 9:**
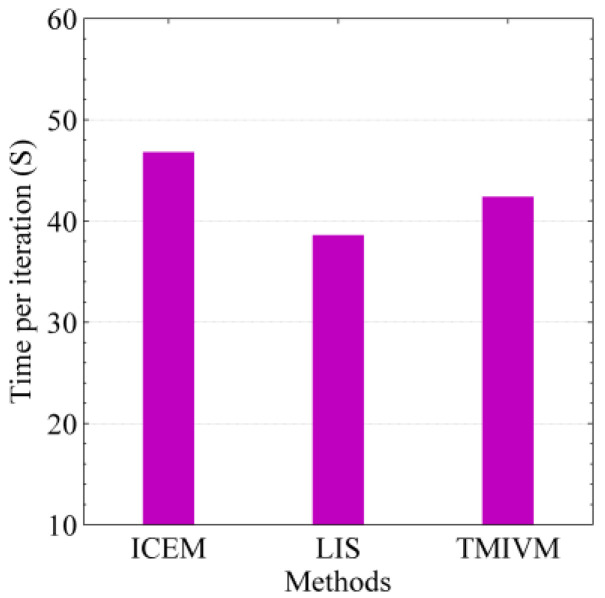
Efficiency of the algorithm in the real data set.

According to the implementation code of the method in the synthetic data set, under the same running environment, we tested the running times of the different methods using the real data set. For the three iterative algorithms, the time needed to execute a single iteration was tested, as shown in Figure 9. Unlike the results in Figure 6, the ICEM method took the most time for one iteration, while the LIS and TMIVM methods took less time. This is because, in each iteration, the ICEM method needed to calculate 341,652 parameters, while the other two methods only needed to calculate 21,588 parameters. When the number of users in the social network data set increases, the number of relationships between users increases exponentially, and the computational advantage of the proposed method becomes increasingly more obvious.

## 5. Conclusions

In this paper, we proposed a method to calculate the mutual influence among users at the topic level, mainly by establishing an influence vector and a susceptibility vector for each user. The dimension of the vector represents the different topic categories. The topic related to the fact that a mutual influence can be simply obtained by multiplying the influence vector elements with the susceptibility vector elements of the corresponding dimension. At the same time, combined with the topic distribution of information, we constructed a method to calculate the propagation probability of specific information in social networks. Then, we used Survival Analysis to establish the likelihood function of cascade records from the perspective of users. Finally, we used the classic gradient descent method to optimize the solution. Compared to existing modeling methods, which take the relationship edge between users as their parameters, the efficiency of this method in parameter training is obvious. Furthermore, the method for modeling the relationships between users considers the communication behavior of information among users to be mutually independent, which is unreasonable since all the communication paths related to the same user have a certain dependence. The vectorized influence and susceptibility model constructed by TMIVM can express this association when calculating the propagation probability, thereby improving the accuracy of information dissemination analysis. The topic carried by the information is a direct factor in promoting the social behavior or discussion of users. The main feature of this method is its ability to integrate the content analysis of informational text, which can effectively capture the differences of information communication in different topics, making the cascade communication analysis more realistic. The calculation of propagation probability based on a vector representation of influence and susceptibility is an important application of the quantitative analysis of mutual influence in the field of communication. It can effectively overcome the over-fitting problem, as well as help in the dynamic analysis of information transmission. The large number of experimental results using the synthetic data set and the real Sina Weibo data set show that the performance of this method is better than that of other comparative methods for propagation path prediction, user forwarding behavior analysis, propagation cascade size estimation, etc., thereby demonstrating the effectiveness of this method. In terms of computational efficiency, with an increase in the number of users and the number of relationships between users in the social network data set, the method presented in this paper takes less time for a single iteration, thus highlighting its computational efficiency.

## Figures and Tables

**Table 1:** Symbol definitions and explanations.

Symbol	Explanation
G	Social Networks
V	A set of users
E	A set of following relationships between users
K	Number of topics
m	An original blog message
$m_{o}$	Topic distribution vector of information m
$C^{m}$	Cascade recording of information m’s propagation
$P_{≼u_{N}}^{m}$	The parent node of $u_{N}$ in information m’s cascade
I	Influence vector
S	Susceptibility vector
$MI_{u\to v}$	Mutual influence of user u on user v
$D_{u\to v}^{m}$	Probability of information m being propagated to v by user u

**Table 2:** Predicted AUC values in the forest fire model.

Cascade Scale	Comparison Method			
	ICEM	NETRATE	LIS	TMIVM
1000	0.539 ± 0.028	0.561 ± 0.024	0.612 ± 0.021	0.685 ± 0.015
2000	0.558 ± 0.033	0.597 ± 0.037	0.651 ± 0.017	0.734 ± 0.014
5000	0.603 ± 0.031	0.647 ± 0.018	0.715 ± 0.015	0.788 ± 0.008

**Table 3:** Predicted AUC values in the Kronecker graph model.

Cascade Scale	Comparison Method			
	ICEM	NETRATE	LIS	TMIVM
1000	0.525 ± 0.045	0.554 ± 0.031	0.568 ± 0.027	0.653 ± 0.019
2000	0.547 ± 0.041	0.589 ± 0.026	0.617 ± 0.028	0.699 ± 0.012
5000	0.596 ± 0.039	0.638 ± 0.021	0.679 ± 0.022	0.741 ± 0.010

**Table 4:** Description of the microblog data set.

Attribute Description	Statistics
User	10,794
Number of following relationships	341,652
Original microblogs	57,347
Retweets	2,892,451
